# Effects of lactic acid bacteria isolated from Tibetan chickens on the growth performance and gut microbiota of broiler

**DOI:** 10.3389/fmicb.2023.1171074

**Published:** 2023-07-20

**Authors:** Lei Wang, Zhengrong Lin, Mahboob Ali, Xiaohui Zhu, Yu Zhang, Siyuan Li, Kun Li, Fareeda Kebzhai, Jiakui Li

**Affiliations:** ^1^College of Veterinary Medicine, Huazhong Agricultural University, Wuhan, China; ^2^Department of Health, Rural Health Center Akhtarabad, Okara, Pakistan; ^3^Institute of Traditional Chinese Veterinary Medicine, College of Veterinary Medicine, Nanjing Agricultural University, Nanjing, China; ^4^Directorate Planning and Development, Livestock and Dairy Development Department Balochistan, Quetta, Pakistan

**Keywords:** Tibetan chicken, growth performance, microbiome, broiler, *Lactobacillus*

## Abstract

Lactic acid bacteria (LAB) are organic supplements that have several advantages for the health of the host. Tibetan chickens are an ancient breed, which evolve unique gut microbiota due to their adaptation to the hypoxic environment of high altitude. However, knowledge of LAB isolated from Tibetan chickens is very limited. Thus, the purpose of this study was to assess the probiotic properties of *Lactobacillus Plantarum* (LP1), *Weissella criteria* (WT1), and *Pediococcus pentosaceus* (PT2) isolated from Tibetan chickens and investigate their effects on growth performance, immunoregulation and intestinal microbiome in broiler chickens. Growth performance, serum biochemical analysis, real-time PCR, and 16S rRNA sequencing were performed to study the probiotic effects of LP1, WT1, and PT2 in broiler chickens. Results showed that LP1, WT1 and PT2 were excellent inhibitors against *Escherichia coli* (*E. coli* ATCC25922), meanwhile, LP1, WT1, and PT2 significantly increased weekly weight gain, villus height, antioxidant ability and gut microbiota diversity indexes in broilers. In addition, LP1 and PT2 increased the relative abundance of *Lactobacillus* and decreased *Desulfovibrio* in comparison with T1 (control group). Additionally, oral LAB can reduce cholesterol and regulate the expression of tight junction genes in broiler chickens, suggesting that LAB can improve the integrity of the cecal barrier and immune response. In conclusion, LAB improved the growth performance, gut barrier health, intestinal flora balance and immune protection of broiler chickens. Our findings revealed the uniqueness of LAB isolated from Tibetan chickens and its potential as a probiotic additive in poultry field.

## Introduction

The intestinal tract contains billions of microbial species, including bacteria, fungi, protozoa, and viruses (Qin et al., 2010). Microbes in the gastro-intestinal tract (GIT) are very important for the health and growth of the host, particularly when it is young. During this important time of exposure to microbes, microbes help the host’s immune system development, nutritional absorption, growth performance and inhibition of pathogenic bacteria (Belkaid and Hand, 2014; Hakansson et al., 2015; Chen et al., 2017). Evidence shows that this sort of alternation of gut microbiota (often called dysbiosis) cause all sorts of health issues including diarrhea and inflammation (Cerf-Bensussan and Gaboriau-Routhiau, 2010; Nagalingam et al., 2011; Arfken et al., 2020; Rutkowski et al., 2022; Uthaibutra et al., 2023). Overall, the balance of the intestinal microbiota is critical for maintaining the health of intestine and regulating the growth of the animal (He et al., 2022; Hu et al., 2023).

Probiotics, which contain organic acids, digestive enzyme activity, immune and antioxidant factors, is considered an effective strategy for the prevention of environmental side effects (Kumar et al., 2022). The potential role of probiotics in promoting physical health is accomplished by the maturation of intestinal barrier and the balance of gut microbiota (de Vos et al., 2022). Previous studies have suggested that probiotic feeding is beneficial for growth performance, immune maturation and gut health (Liu B. et al., 2022). *Lactic acid bacteria* (LAB) are the most important strain of probiotics due to its role in maintaining microbial flora balance and regulating animal growth (Lutful, 2009; Abd et al., 2020). The proposed mechanisms for *Lactobacillus* include bacteriocin and short-chain fatty acid production, which enhance the inhibitory effects against pathogens and promote gut microbiome profiles (Vernocchi et al., 2020). Some of the most commonly recognized genera in the LAB include *Leuconostoc*, *Bifidobacterium*, *Pediococcus*, *Weissella*, *Lactobacillus*, *Lactococcus*, and *Streptococcus* (Karami et al., 2017).

The Tibetan chicken, which are mainly situated in the Tibetan Plateau (elevation of 3,000~5, 000 m), is one of the precious breed with high tolerance to cold and low oxygen levels. Previous studies have reported the probiotic potential of Tibetan animals, including the positive effect of growth of *Lactobacillus* isolated from Tibetan yaks (Wang et al., 2022). It is speculated that the unique environment (high altitude) of the Tibetan Plateau promotes the development of microbiota diversity in the gut of the local inhabitants including animals, which helps in the disease resistance and maintain their health in harsh conditions (Li et al., 2016). It is well established that the population or number of *Lactobacillus* species that can be associated with excellent probiotic protection (Pan et al., 2018). However, there is very little knowledge about the LAB of Tibetan chickens. So, we hypothesized that LAB from Tibetan chickens has the potential to improve broiler performance. Therefore, this study aims to isolate LAB bacteria from Tibetan chickens and investigate their effects on growth performance, immunoregulation and intestinal microbiome in broiler chickens. This study highlights the beneficial effects of LAB on broiler chickens, indicating that LAB can be used as a probiotic additive in broiler feed.

## Materials and methods

### Isolation and identification of LAB

Cecum content from 59 Tibetan chickens was collected in Linzhi, Tibet, and immediately frozen in liquid nitrogen and stored at −80 until analysis. Briefly, 1 g cecum content was added into MRS broth and shaken (200r/h) for 24 h at 37°C. The 10 μL supernatant was incubated on MRS agar (Hangzhou Reagents, China) at 37°C for 48 h in an anaerobic condition. The creamy, mellow roundness was suspected to be LAB and selected for purification. This process was repeated two to three times until a single pure colony of the same form was obtained. A suspected LAB colony was then selected and inoculated in MRS broth for 24 h.

The isolated strains were identified by Gram-staining, biochemical tests and 16S rRNA sequencing. Microbial genomic DNA was extracted using the method described previously (Wang et al., 2018) and 16S rRNA gene was amplified using universal PCR primers 27F (5 0 -AGAGTTTGATCCTGGCTCAG-3 0) and 1492R (5 0 -TACGGCTACCTTGTTACGACTT-3 0). The thermal cycling parameters were as follows: 95°C for 5 min followed by 35 cycles of denaturation at 94°C for 1 min, primer annealing at 54°C for 45 s, elongation at 72°C for 1 min and thermal retardation at 72°C for 10 min. Furthermore, the PCR products were sequenced at the Qingke Biotech Company (Wuhan, China) and analyzed by using nucleotide BLAST on the NCBI website.

### Ability of LAB to tolerate acid and cholate

With the purpose of testing the survival rate of LAB strains in acidity and bile salt environment. 100 μL bacterial solutions (1 × 10^8^ CFU/mL) were inoculated in MRS medium with different pH (pH = 2, 3, 4, 5, 6) or bile salt levels (i.e., 0.1, 0.2, 0.3, 0.4, 0.5%) for 18 h. A culture medium of 100 μL was then spread on a MRS agar plate and incubated in an anaerobic condition for 24 h. The survival equation was consistent with the method described previously (Wang et al., 2018). Strain survival rate (%) = [E/C] × 100%, E is the number of colonies in the experimental group, C is the number of colonies in the control group. Non-bile salted MRS and non-HCl MRS were used as controls.

### *In vitro* antibacterial test

The anti-*E. coli* ability of LAB was investigated by the diffusion test. First, we got a 5 mm hole with a punch in Luria-Bertani (LB) agar planted with 100 μL *E. coli* ATCC 25922 (1 × 10^8^ CFU/mL). Then, 100 μL LAB culture fluid (1 × 10^8^ CFU/mL) was injected into the 5 mm hole. Blank disks were inoculated with 100 μL MRS medium. The inhibitory zone of *E. coli* was measured after incubation at 37°C for 24 h. At the same time, the drug-sensitive test was carried out to explore the difference between LAB and antibiotics for *E. coli*.

### Animal experiments

Eighty, 1 day healthy Arbor Acres (AA) chickens with similar initial weight (50 ± 3 g) were purchased from a commercial hatchery (Jingzhou, China) and housed under a standard feeding environment (humidity: 62%, temperature: 32°C, illumination time: 23 h/1 h light/dark cycle) in Huazhong Agricultural University’s animals’ facility. The experiment started after 3 days of acclimation of broiler chickens and continued until the end of 28 days. On the fourth day, all broiler chickens were randomly distributed into four groups (*n* = 20) named T1 (control group), T2, T3 and T4. The T2, T3, and T4 groups were supplied with the LP1 solution (1 × 10^8^ CFU/mL), WT1 solution (1 × 10^8^ CFU/mL) and PT2 solution (1 × 10^8^ CFU/mL), respectively. All broiler chickens had free access to water and feed during the 28-day trial. Weekly records of body weight during the early and late stages. At 28 days, all chickens were anesthetized before slaughter. Cecum contents were collected for gut microbial diversity, and 2 cm cecum tissues were preserved in a formaldehyde solution (4%) to observe the appearance and morphology of tissue sections with reference to He’s method (He et al., 2022). The formaldehyde-treated cecal tissues were embedded in paraffin, sliced and stained with H&E. In addition, the morphology of each tissue slice and the height of the intestinal villi were observed and measured using light microscopy.

### Biochemical detection

3 mL blood was collected from the jugular vein into a vacuum blood collection tube and centrifuged at 3000 r/min for 30 min to obtain the serum. Serum biochemical indices were detected using biochemical kits according to the manufacturer’s procedures. All kits were purchased from Nanjing Jiancheng Institute of Biological Engineering (Nanjing, China). Serum biochemical indices including Serum total antioxidant capacity (T-AOC), triglyceride (TG), low density lipoprotein cholesterin (LDL-C), high density lipoprotein cholesterin (HDL-C), catalase (CAT), glutathione peroxidase (GSH-Px), superoxide dismutase (SOD) and malondialdehyde (MDA) were measured for further investigation.

### RNA extraction and real-time PCR

With reference to Zhang’s method (Zhang et al., 2022), RNA was extracted from 2 g cecum tissue by TRIzol reagent (Accurate Biotechnology Co., Ltd), and gDNA was obtained by reverse transcription according to Yu’s method (Yu et al., 2017). The expression levels of inflammatory factors (IL-1β, IL-6, IL-10, TLR 4) and tight junction cytokine (ZO-1) were measured using RT-PCR. 10 μL of PCR reaction system containing 1 μL of cDNA, 3.6 μL of DEPC water, 0.4 μL of each primer and 5 μL of mix (Vazyme, Nanjing, China). The thermal cycling parameters were 95°C for 2 min, annealing at 95°C for 20 s and 40 cycles of extension at 60°C for 30 s, at the end, annealing at 72°C for 2 min. The relative expression of each gene was calculated based on the expression of reference gene of β-actin gene.

### DNA extraction and 16S rRNA high-throughput sequencing

The genomic DNA was extracted by the QIAamp DNA Mini Kit (QIAGEN, Hilden, Germany) and quantified using a UV–visible spectrophotometer (NanoDrop 2000, United States). With the aim to amplify the V3/V4 region of 16S rRNA, the primers (338F: ACTCCTACGGGAGGCAGCA and 806R: GGACTACHVGGGTWTCTAAT) were synthesized. The 2% agarose gel electrophoresis and the AxyPrep DNA gel Extraction Kit (Axygen, CA, United States) were used to evaluate the PCR amplified products and retrieve the target fragment, respectively. The samples were mixed proportionally to the corresponding reference qPCR results and the sequencing volume. The qualified PCR products were used to construct sequencing libraries by TruSeq Nano DNA LT Library Prep Kit (Illumina, CA, United States). At the same time, the final fragments were selected and purified using electrophoresis on a 2% agarose gel. Eventually, eligible libraries were sequenced for high-throughput sequencing according to Luo’s method (Liu S. et al., 2022).

### Bioinformatics and statistical analysis

Raw data from high-throughput sequencing was qualitatively screened using QIIME software (QiIME 1.9.1). According to the barcode information, the qualified sequences were classified and clustered into OTUs based on 97% similarity. Meanwhile, representative sequences were identified and phylogenetic analysis was conducted. The relative abundance distribution of OTUs in each sample was used to calculate gut microbial diversity. β diversity was used to analyze the differences and similarities of the main components of intestinal flora. More precisely, scattered curves are generated for each sample in order to evaluate sequencing depth.

## Results

### Colony morphology and genetic analysis

**Figure 1:**
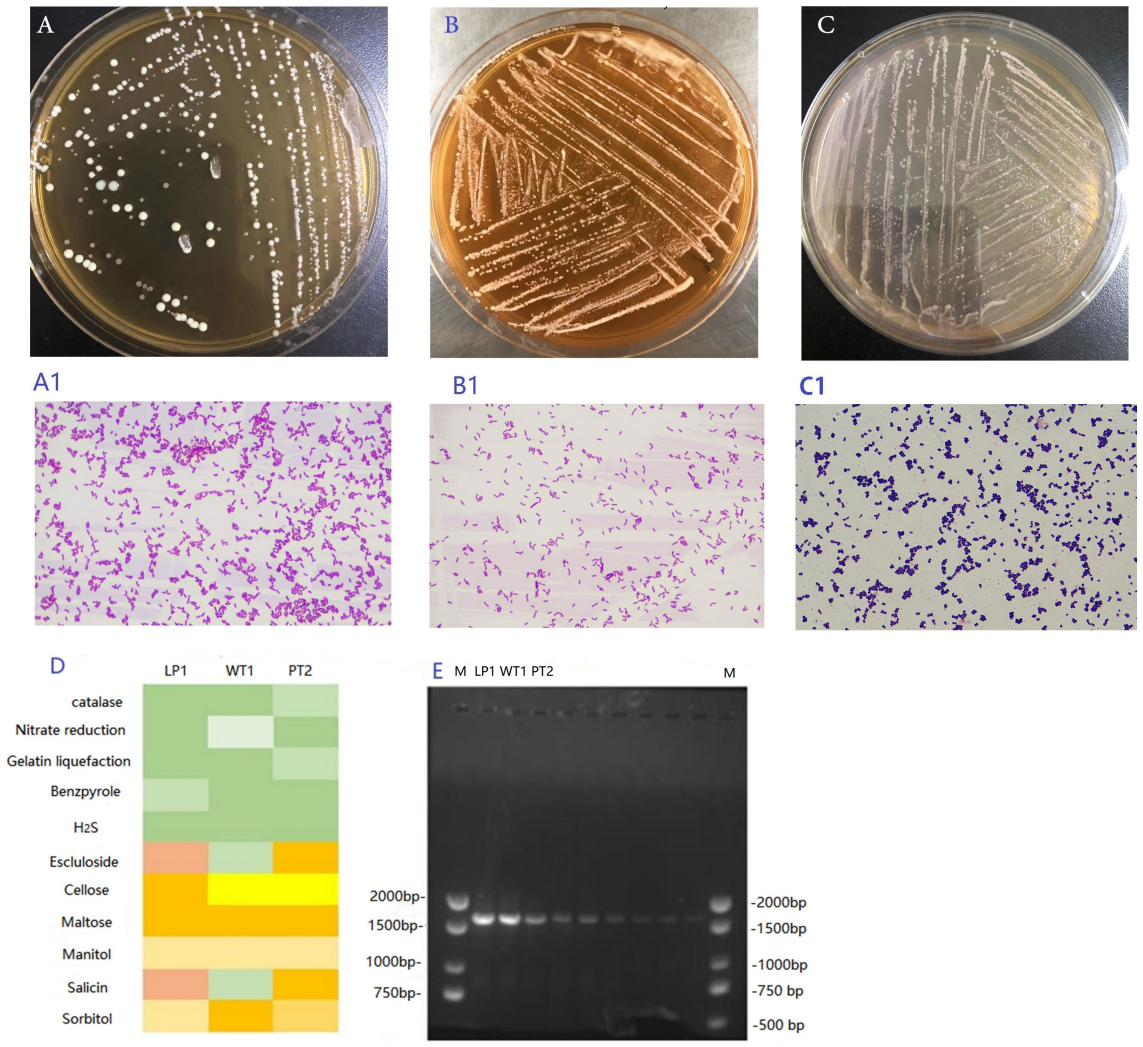
**(A–C)** The colony morphology of the LP1 **(A)**, WT1 **(B),** and PT2 **(C)**. **(A1–C1)** The Gram staining result of LP1 **(A1)**, WT1 **(B1)**, and PT2 **(C1)**. **(D)** The biochemical result of LP1, WT1 and PT2. **(E)**The 16S rRNA agarose gel electrophoresis of LP1, WT1, PT2 PCR amplified product. M: 2,000 bp DNA marker.

The colonies were milky white, round and smooth (Figures 1A–C). *Lactobacillus plantarum* (LP1; Figure 1A), *Weissella criteria* (W. criteria; Figure 1B), and *P. pentosaceus* (PT2; Figure 1C) were Benzpyrole-negative, H_2_S-negative, Maltose-positive and own other unique biochemical characteristics (Figure 1D). Besides, LP1 (Figure 1A1) and WT1 (Figure 1B1) were Gram-positive bacilli with short rod-like morphologies, the PT2 was Gram-positive coccus and looks spherical and small (Figure 1C1).

The 16S rRNA amplification product was detected by 1% agarose gel electrophoresis (Figure 1E). The length of the 3 amplified fragments was about 1,500 bp (Figure 1E), which was in accordance with our expected value. Additionally, the gene sequencing analysis revealed that WT1 was 99% homologous to *W. criteria*. PT2 manifested 99% homologous to *P. pentosaceus*, and LP1 showed 99% homologous to *L. plantarum.*

### Ability of *Lactobacillus* to tolerate acid and bile

**Figure 2:**
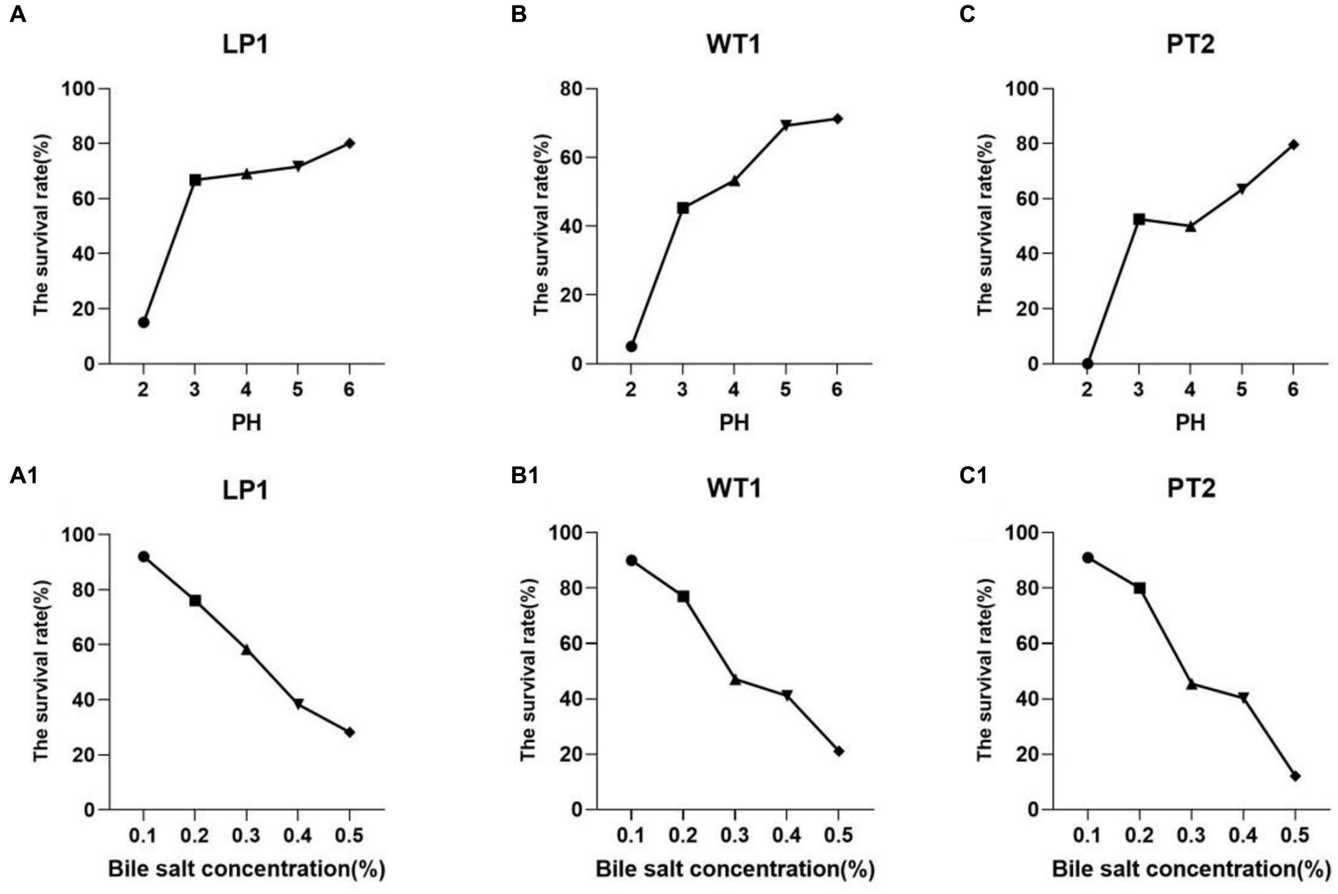
**(A–C)** The tolerance of the LP1, WT1, and PT2 to acid condition [**(A)** LP1, **(B)** WT1, **(C)** PT2]. **(A1–C1)** The tolerance of the LP1, WT1 and PT2 to bile salt environment [**(A1)** LP1, **(B1)** WT1, **(C1)** PT2].

The isolated strains were incubated at different pH (2, 3, 4, 5, 6) or bile salt (0.1%, 0.2%, 0.3%, 0.4%, 0.5%) for 16 h at 37°Cfor 16 h. All the strains *Lactobacillus Plantarum* (LP1), *Weissella cibaria* (WT1), and *Pediococcus pentosaceus* (PT2) manifested to be tolerant to strong acid (pH = 3; Figures 2A–C), such as, the survival rate of LP1, WT1, and PT2 reached 66.78%, 45.29%, and 52.54% at pH = 3, respectively. Meanwhile, at 0.3% bile salt, the survival rate of 3 strains was observed as LP1:58.38%, WT1: 47.13%, PT2:45.45%. In particular, LP1 showed great tolerance to the bile salt, reaching a survival rate of 58.38% (Figures 2A1–C1).

### Antibacterial effect of *Lactobacillus* from Tibetan chickens

**Figure 3:**
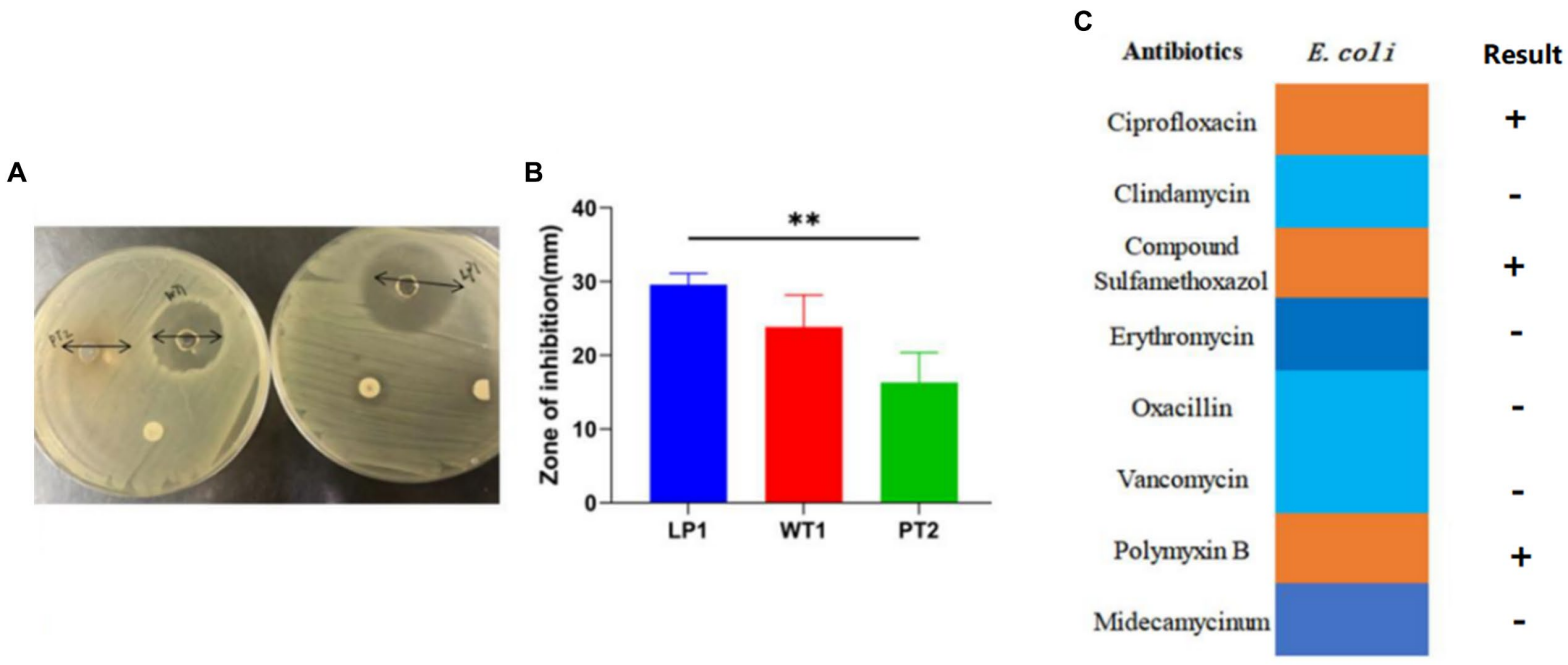
**(A)** The antibacterial effect of LP1, WT1, and PT2. **(B)** The inhibition zones diameter of LP1, WT1, PT2 against *Escherichia coli* ATCC25922. **(C)** Antibiotic susceptibility test for *E. coli* ATCC25922. ** represents *p* < 0.01.

The obtained results revealed that LP1, PT2, and WT1 had positive anti-*E. coli* effect. The inhibitory zone diameter ranged from 14 mm to 31 mm (Figure 3A), Notably, the average *E. coli* ATCC25922 growth inhibition capability of LP1 reached 30.61 mm (Figure 3B), showing a more considerable effect than PT2 and other 7 kinds of antibiotics (*p* < 0.01; Figure 3C).

### Effect of LAB on broiler growth performance

**Figure 4:**
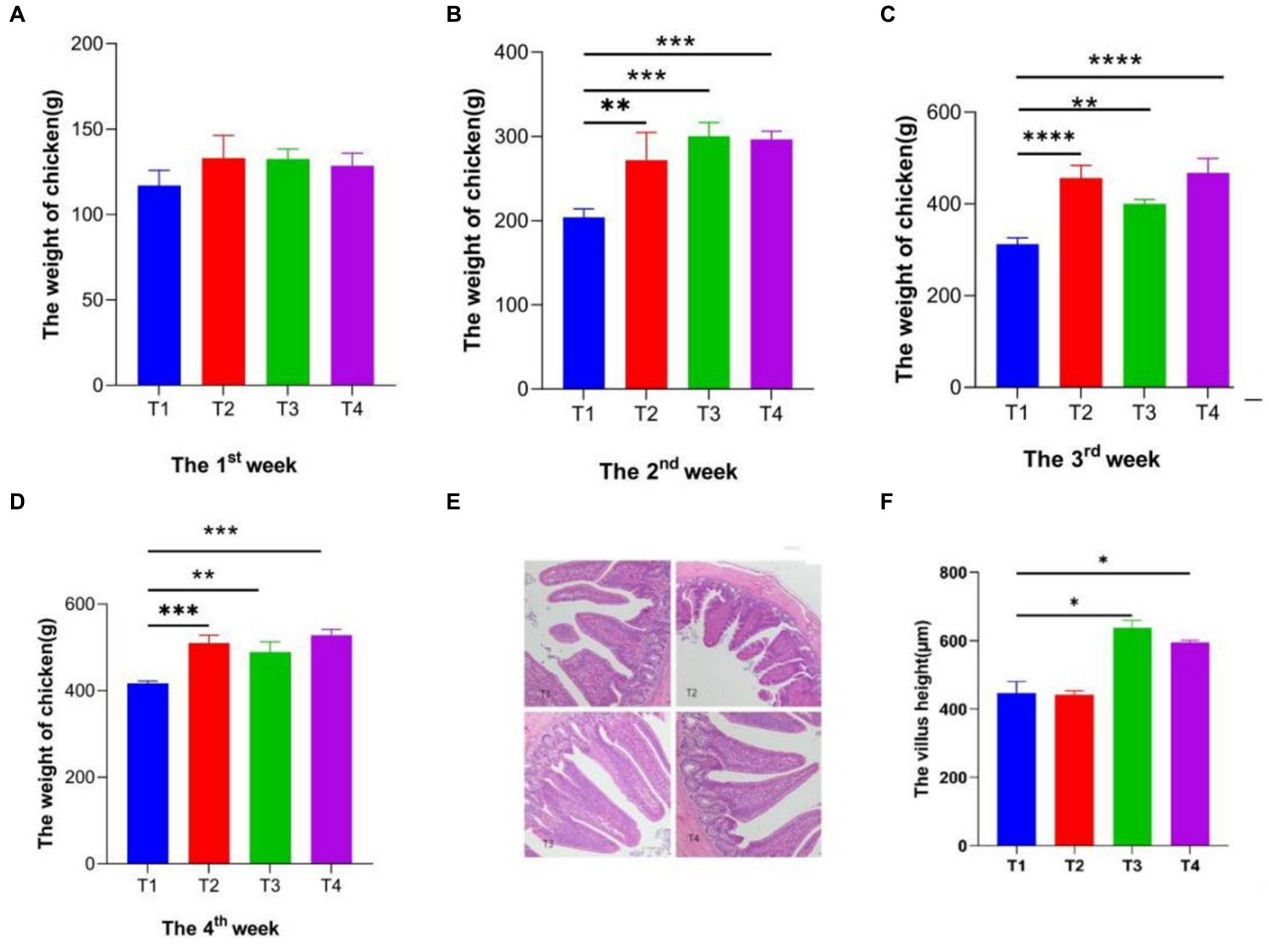
**(A–D)** The weekly weight of Broiler chickens per group, T1: Control group; T2: LP1 supplement group; T3: WT1 supplement group; T4: PT2 supplement group. **(E,F)** The morphology of cecum villus and villus height. * represents *p* < 0.05, ** represents *p* < 0.01, *** represents *p* < 0.001, **** represents *p* < 0.0001.

In the current study, it was found that administration of LAB (1 × 10^8^ CFU/mL) could significantly improve the growth efficiency and weight gain of AA broilers. The data clearly showed that feeding LP1, WT1, and PT2 accelerated weight gain in AA broilers. During the first week, there was no obvious difference between the groups (Figure 4A). While, the weight difference of broilers gradually increased with the extension of feeding time. In the second week (Figure 4B), there was a significant difference among the T1 (control), T2 (LP1 supplement), T3 (WT1 supplement), and T4 (PT2 supplement) groups. Especially, LP1 manifested a perfect effect on weight gain during week 3 compared to the T1, T3 groups (Figure 4C). At week 4 of feeding (Figure 4D), the mean body weight of the T2, T3 and T4 groups was 510.12 g, 489.66 g and 528.32 g, respectively. T2 (510.12 g) and T4 (528.32 g) were obviously higher than T1 group (416.66 g; *p* < 0.0001). The results implied that in terms of body weight gain, LP1 and PT2 have extensive potential and great exploitation prospects in poultry feed.

### Effects of *Lactobacillus* on villus height in the small intestine of AA broilers

It was found that the villus morphology of the cecum was clear and intact under the Olympus BX53 microscope (Figure 4E), in which there was no difference in the intestinal epithelial morphology of 4 groups (T1, T2, T3, and T4). Notably, the height of the cecum villi of T3, T4 was obviously higher than T1. Conversely, there was no difference between T1 and T2 groups. In particular, the villus height of the T3 group exhibited a higher surface area compared to other groups. In all, WT1 and PT2 positively promote the growth of cecum villi (Figure 4F).

### Analysis of serum biochemistry

**Figure 5:**
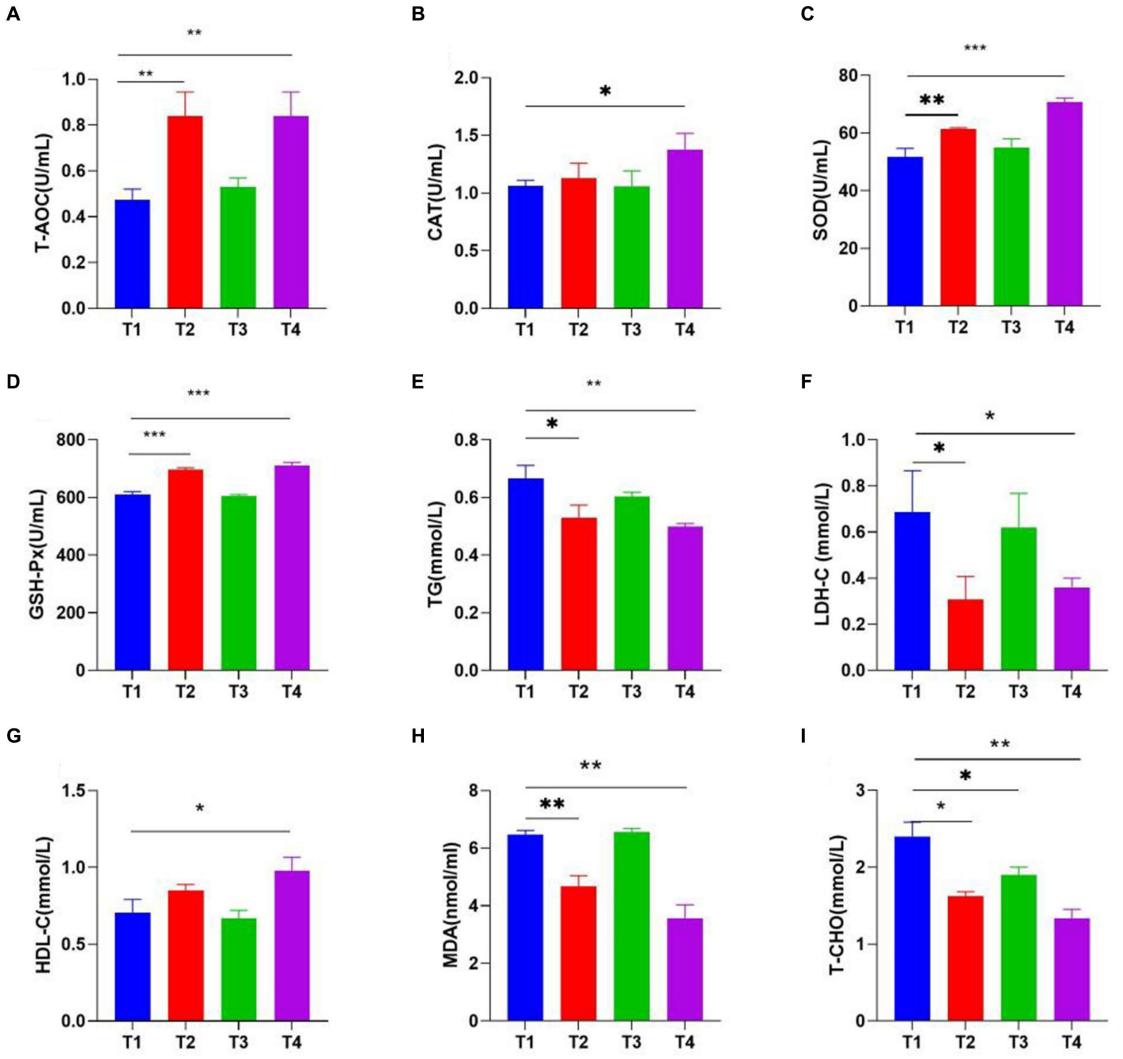
The Effects of LAB (T1: control group, T2: LP1, T3: WT1, and T4: PT2) supplementation on serum biochemical indexes [**(A)** T-AOC, **(B)** CAT, **(C)** SOD, **(D)** GSH-Px, **(E)** TG, **(F)** LDH-C, **(G)** HDL-C, **(H)** MDA, **(I)** T-CHO]. * represents *p* < 0.05, ** represents *p* < 0.01, *** represents *p* < 0.001.

The experimental results showed that LAB effectively improves the antioxidant capacity of broiler chickens, LP1 and PT2 were likely to be effective choices for the improvement of antioxidant capacity of the organism, characterized by increased T-AOC, CAT, GSH-Px, SOD and decreased MDA levels. PT2 significantly reduced serum LDL-C (*p* < 0.01), T-CHO (*p* < 0.01) and TG (*p* < 0.01). At the same time, the concentration of HDL-C in the T4 group was significantly higher than that of control group (Figure 5).

### The inflammatory and tight junction gene expression analysis

**Figure 6:**
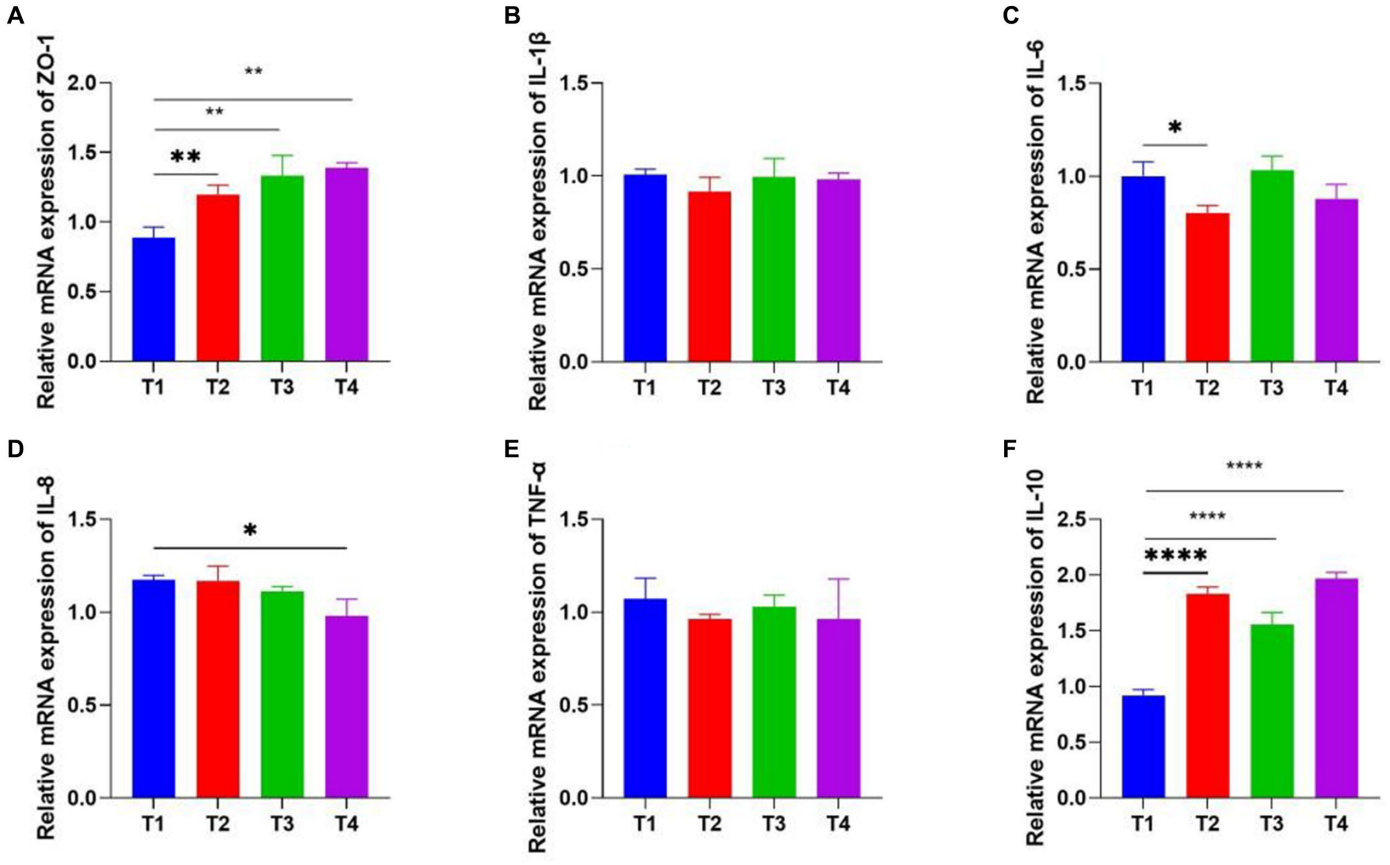
Real-Time PCR analysis of inflammatory and tight junction genes expression. ZO-1 **(A)**, IL-1β **(B)**, IL-6 **(C)**, IL-8 **(D)**, TNF-α **(E)**, IL-10 (**F**; * represents *p* < 0.05, ** represents *p* < 0.01. ****p < 0.0001).

Real-time fluorescence quantitative PCR was performed to detect the levels of inflammatory and tight junction (ZO-1) cytokine production in tissues. ZO-1 cecum concentrations were significantly up-regulated under LP1, WT1, and PT2. The relative mRNA expression of inflammatory cytokines (IL-1β, IL-6, IL-8, TNF-α) was 0.8, 0.85, 1.22, 0.94 in T2 group, 1.12, 1.0, 1.14, 0.89 in T3 group and 1.25, 0.98, 1.07, 0.91 in T4 group, with no statistically significant differences compared to the control group. Interestingly, the anti-inflammatory cytokine (IL-10) concentrations were 1.9, 1.8, and 2.0 in the T2, T3, and T4 groups, respectively, showing significantly higher than those in the control group (Figure 6).

### Comparison of intestinal microbiota diversity

**Figure 7:**
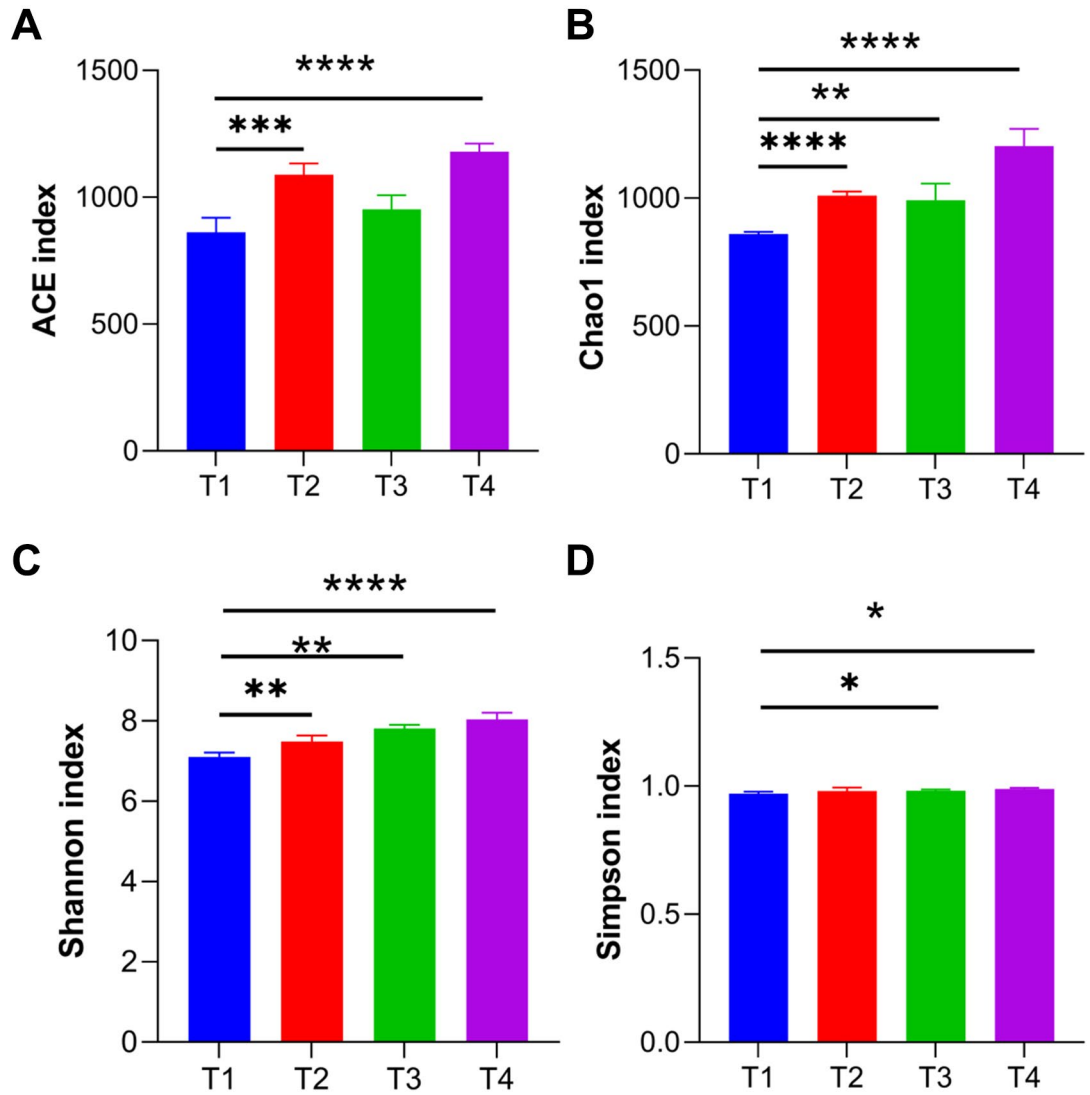
Effects of LP1, WT1, and PT2 on intestinal diversity and abundance. Gut microbial diversity can be assessed by ACE **(A)**, Chao1 **(B)**, Shannon **(C)**, and Simpson **(D)**. * represents *p* < 0.05, ** represents *p* < 0.01, *** represents *p* < 0.001, **** represents *p* < 0.0001.

The 16S rRNA sequence was assigned as an OTU with at least 97% sequence similarity. Alpha diversity analysis detected that there were significant differences in the Chao1 (T1:858.86, T2:999.15, T3: 991.48, T4: 1247.72), ACE (T1: 836.26, T2: 1089.08, T3: 953.26, T4: 1178.26) and Shannon (T1: 6.6, T2: 7.49, T3: 7.82, T4: 8.04; Figures 7A–C). On the other hand, there was no significant difference in Simpson index between T1 and T2 (Figure 7D). Inter-group analysis of alpha diversity intuitively manifested that LAB (LP1, WT1, PT2) supplementation significantly increases broiler’s gut microbiota abundance and diversity.

### Effect of LAB on the gut flora of broilers

**Figure 8:**
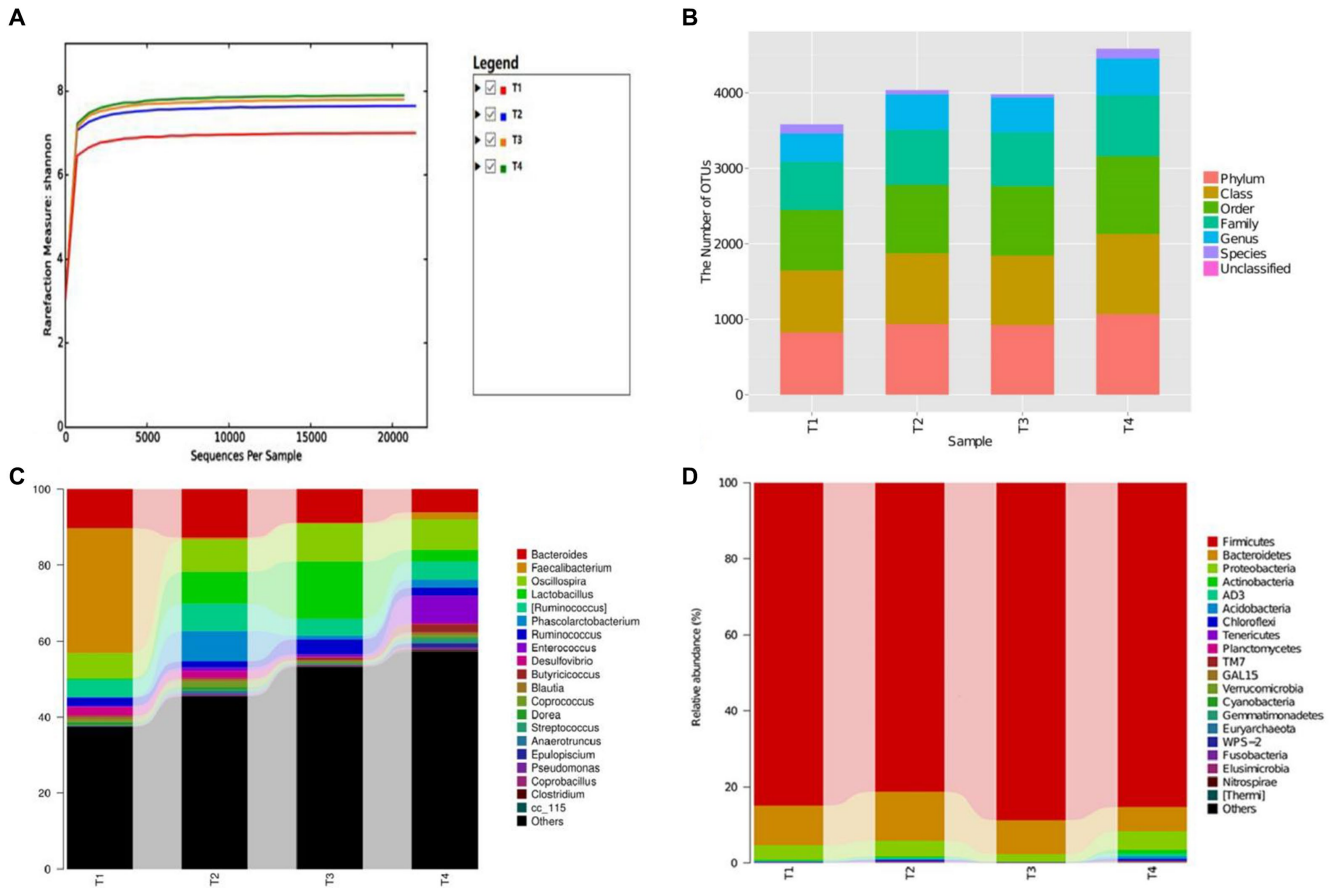
**(A)** The bacterial rarefaction and rank abundance curves were used to assess the quality of the sequencing. **(B)** The result of OTUs classification and identification of each group, **(C,D)** The relative abundance of gut microbial community at the phylum level **(C)** and genus level **(D)**.

**Figure 9:**
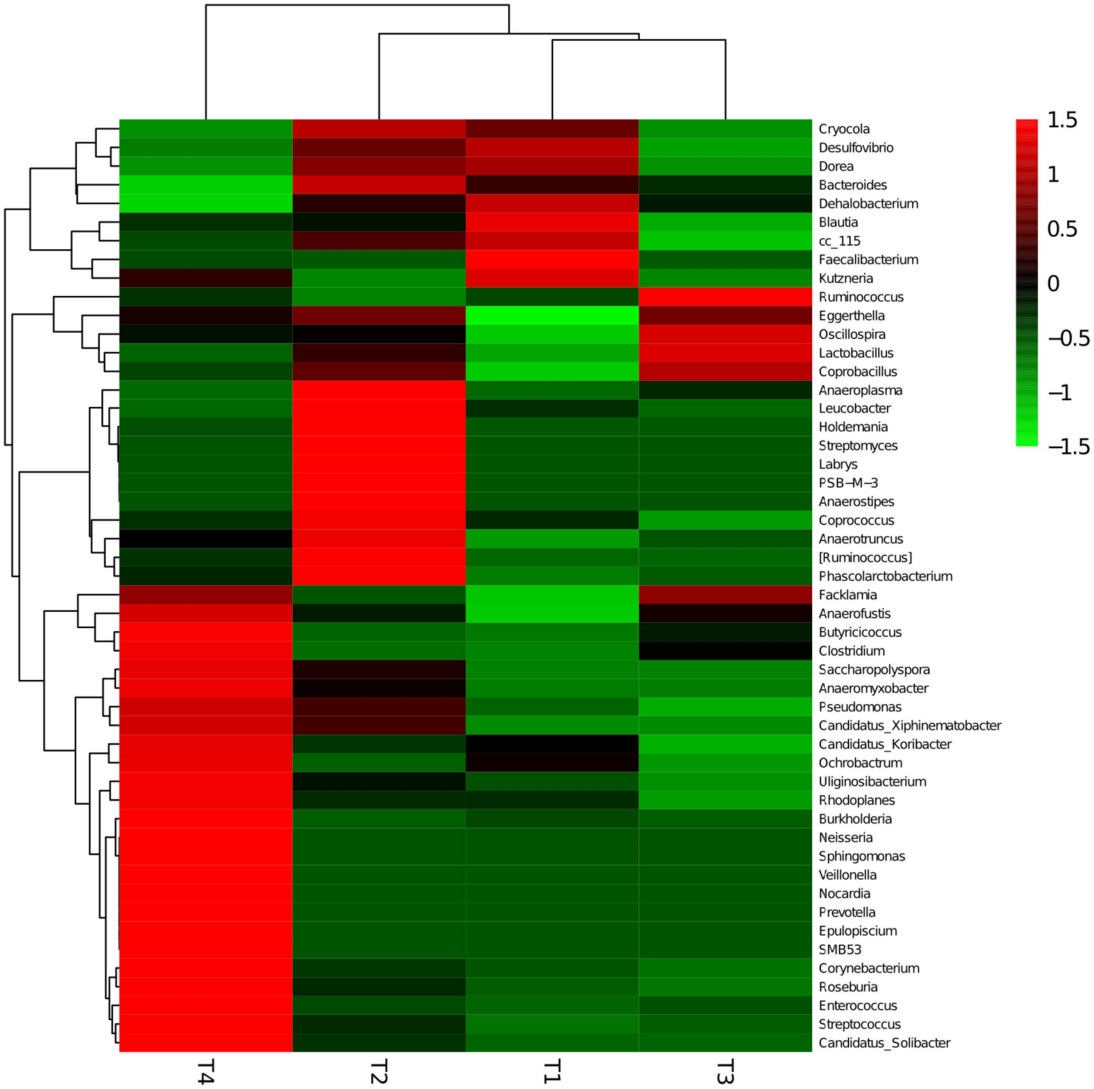
Heatmaps of the top 50 bacterial genera in the T1, T2, T3, and T4 groups, showing the abundance distribution and similarity of bacteria in groups.

Not only was there a significant difference in gut microbiota diversity between groups, but there was also a variation in microbial proportions (Figures 8A,B). Specifically, 14 phyla, 28 taxa, and 73 genera were detected from all samples. At the phylum level (Figure 8C), the *Firmicutes* (84.90%, 81.20%, 88.70%, 85.30%), *Bacteroidetes* (10.50, 12.90, 8.90, 6.50%), *Proteobacteria* (3.8, 4.2, 2.1, 4.9%), *Actinobacteria* (0.2%, 0.3%, 0, 0.9%), *Acidobacteria* (0.1%, 0.3%, 0, 0.7%), *Chloroflexi* (0.1%, 0.2%, 0, 0.4%), *AD3* (0.2%, 0.3%, 0, 0.8%) and *Proteobacteria* (3.8%, 4.2%, 2.1%, 4.9%) were the most abundant phyla in 4 groups, which together constitute more than 98.00% of the gut microbiota. Notably, the *Firmicutes* (85.3%) and *Proteobacteria* (4.9%) were also the predominated community in the T4 group, showing a higher microbial abundance compared to the T1 (*Firmicutes*: 84.90%, *Proteobacteria*: 3.80%). Similarly, *Acidobacteria in* T2 (0.30%) and T4 (0.90%) groups showed higher richness than T1 group (0.2%). At genus level, the top 10 genera were *Clostridiales* (8.6%, 15.8%, 24.5%, 20.8%), *Ruminococcaceae* (20.8%, 15.3%, 19.2%, 13.1%), *Bacteroides* (10.4%, 12.8%, 8.9%, 6.3%), *Faecalibacterium* (32.8%, 0.6%, 0.3%, 1.7%), *Oscillospira* (6.7%, 8.3%, 9.8%, 8.1%), *Lactobacillus* (0.4%, 8.5%, 15%, 3%), [*Ruminococcus*] (4.4%, 7.2%, 4.4%, 4.8%), *Lachnospiraceae* (4.4%, 6.4%, 5.3%, 5.9%), *Ruminococcus* (2.1%, 1.7%, 4.1%, 2.2%; Figure 8D). The highest amount of *Lactobacillus* was detected in the T3 group (T3: 15.00%) compared to other groups (T1: 0.40%, T2: 8.50%, T4:3.00%). A comparison between LAB supplement groups (T2, T3, T4) and the control group (T1) revealed a significant increase in the abundance of *Clostridiales*, *Ruminococcus*, *Lachnospiraceae*. Conversely, the pathogen of *Desulfovibrio* (2.10%) was a dominant bacterium in T1 group, which was higher than that in LAB supplementary treatment groups (T2: 1.80%, T3: 0.20%, T4:0.40%). Moreover, the gut microbiota distribution and richness among groups was revealed using heatmap (Figure 9). The experimental results largely revealed that LAB (LP1, WT1, PT2) complementary therapy can regulate and enrich the gut microbial diversity. Cumulative data from *in vitro* and *in vivo* analyses suggest that *L. plantarum*, *W. criteria*, and *P. pentosaceus* may be worthy probiotics for chicken feed.

## Discussion

Probiotics are often used as green and healthy ingredients in broiler breeding, which have been reported to promote growth performance, gut morphology, immunity mature (Diaz et al., 2019). The dietary of probiotics was regarded as “Generally Recognized as Safe (GRAS)” alternative to antibiotics (Buntyn et al., 2016).

While there have been many studies on the probiotic effects of LAB in broiler chickens, few studies have been available on LAB isolated from Tibetan chickens. Potential probiotics must have an excellent survival rate in acidic and bile salt environments in the digestive tracts (Khalil et al., 2018). LP1, WT1, and PT2 showed great resistance activity to acid (pH = 3) and bile salt (0.3%), also have strong antibacterial property to *E. coli*. Previous studies have shown that *Lactobacillus* help prevent *E. coli* infections (Makhloufi et al., 2013). In T. Benbara’s reported, *L. plantarum* might be a good alternative to antibiotics in birds feeding (Benbara et al., 2020). Similarly, Boris et al. reported that *Lactobacilli* have the ability to inhibit pathogens including *E. coli*, *P. aeruginosa*, *S. aureus*, and *Salmonella typhimurium* (Boris et al., 2001), which is in accordance with our results. The results suggested that a diet rich in LAB may lower the risk of *E. coli* infection.

In addition, a detailed assessment of LAB’s antioxidant capacity, anti-inflammatory properties and immunity ability was found in the study, leading to the discovery of a relationship between LAB and broilers. LP1, WT1, PT2 exposure upregulated ZO-1 levels, also, pro-inflammatory factors were significantly down-regulated and anti-inflammatory factors were up-regulated in the presence of LP1, showing that LP1 had protective effects on intestinal barrier function and mucosal immunity, LP1 has great potential as an effective source of inhibiting and relieving inflammation, which in agreement with previous researches (Mandal et al., 2014; Bakkeren et al., 2019; He et al., 2022).

The intestinal villi have many structural functions that aid nutrient absorption. Previous studies have reported that probiotics contribute to the development of the intestinal villi and gut integrity, which promotes nutrient absorption (Li et al., 2018; Soumeh et al., 2021). This is why probiotics promote weight gain and gut health. The inclusion of WT1 and PT2 improved the height of intestinal villi and gut integrity in broilers. These findings are consistent with previous reports that probiotics improve intestinal health in chickens through villi development (Pimentel and Chan, 2007; Linninge et al., 2019; Zhang et al., 2021).

Balanced and stable gut microbiota is a prerequisite for complex gut activity that protects against infection. Alteration of the homeostatic balance will inevitably lead to metabolic disorders (Wang et al., 2020). Additionally, the decline in the richness of gut microbiota was associated with chronic diseases including diarrhea and inflammation (Zhang et al., 2021). Early research showed that gut microbiota diversity is constantly related to various environmental and weather conditions (Del et al., 2022). LP1, WT1 and PT2 manifested a significant improvement on gut microbial diversity and abundance. Similarly, Linninge et al. (2019) and Tan et al. (2020) both confirmed for us the view that *Lactobacillus* restored the balance and diversity of gut microbiota.

Interestingly, while the species of the dominant phyla did not change, the rates did. Compared to T1, there was a significant increase in the proportion of *Fimicutes* and *Actinobacteria* in the LAB complement group. At the genus level, *Lactobacillus*, *Enterobacteriales* and *Bacillales* in groups T2, T3, and T4 show dramatic increases compared to T1. For example, *Lactobacillus* species T2, T3 and T4 had rates of 9.6, 15.9 and 11.1% respectively, however, T1 has a rate of 0.1%. Most of the bacteria that increased in the T2, T3, and T4 groups, including *Faecalibacterium* and *Oscillospira*, are considered beneficial probiotics due to their anti-inflammatory and immunoregulatory properties. Elham A. Soumeh also found that the abundance of *Lactobacillus* was significantly increased in *Lactobacillus*-induced broiler chickens, which was in line with our finding (Soumeh et al., 2021). On the other hand, LAB suppresses the production of pathogens. Similarly, Teruo Urata et al. found that *Desulfovibrio* species increases the risk of infection (Urata et al., 2008). Comparable results were also found in the present study, where the fraction of *Desulfovibrio* species in group T1 is significantly higher than in other groups. The reason for this result is that the WT1 and PT2 likely inhabit the growth of *Desulfovibrio*. In line with our findings, both Cean et al. (2015) and Wang et al. (2018) observed that LAB had an inhibitory effect against Gram-negative pathogens.

Our results demonstrated that the growth performance and gut microbiota balance of the broilers were markedly improved by LP1, WT1, and PT2 isolated from Tibetan chickens, which also exhibited antibacterial, anti-inflammatory, and immunomodulatory properties. These results indicated that the strains LP1, WT1, and PT2 isolated from Tibetan chickens were good candidates for probiotics.

## Conclusion

Overall, the present study characterized dynamic changes in growth performance and gut microbial communities in broilers treated with LAB isolated from Tibetan chickens. The results show that LAB including *L. plantarum* and *P. pentosaceus* significantly contribute to weight gain, antioxidant ability and gut microbial diversity in broiler chickens. Administration of PT2 to broilers resulted in reduced cholesterol and helped maintain the integrity of the cecum barrier. Furthermore, LP1, WT1 and PT2 successfully inhibited colonization and survival of *E. coli* ATCC25922. These baseline results illustrate the rarity and uniqueness of Tibetan chicken in another perspective. Moreover, our research data from *in vitro* and *in vivo* analyses indicated that LP1, WT1 and PT2 isolated from Tibetan chicken might be the potential probiotics if used in broiler feed.

## Data availability statement

The datasets presented in this study can be found in online repositories. The original sequence data was submitted to the Sequence Read Archive (SRA; NCBI, United States) with the accession no. PRJNA954678.

## Ethics statement

The animal study was reviewed and approved by the Ethics Committee of Huazhong Agricultural University.

## Author contributions

LW: experimental design, experiment, and writing—original draft. MA, KL, and FK: editing and reviewing. ZL and SL: investigation and animal experiment. XZ and YZ: experiment. JL: editing and funding. MA: drafting the work and critically revising. FK: critical revision. All authors contributed to the article and approved the submitted version.

## Funding

The study was supported by The Tibet Autonomous Region Science and Technology Department 2023 Key Research Development Program (XZ202301ZY0016N).

## Conflict of interest

The authors declare that the research was conducted in the absence of any commercial or financial relationships that could be construed as a potential conflict of interest.

## Publisher’s note

All claims expressed in this article are solely those of the authors and do not necessarily represent those of their affiliated organizations, or those of the publisher, the editors and the reviewers. Any product that may be evaluated in this article, or claim that may be made by its manufacturer, is not guaranteed or endorsed by the publisher.

## References

[ref1] Abd E. M. El-Saadony M. T. Shafi M. E. Qattan S. Batiha G. E. Khafaga A. F.. (2020). Probiotics in poultry feed: a comprehensive review. J Anim Physiol Anim Nutr 104, 1835–1850. doi: 10.1111/jpn.13454, PMID: 32996177

[ref2] Arfken A. M. Frey J. F. Summers K. L. (2020). Temporal dynamics of the gut bacteriome and mycobiome in the weanling pig. Microorganisms 8:868. doi: 10.3390/microorganisms8060868, PMID: 32526857 PMC7356342

[ref3] Bakkeren E. Huisman J. S. Fattinger S. A. Hausmann A. Furter M. Egli A.. (2019). Salmonella persisters promote the spread of antibiotic resistance plasmids in the gut. Nature 573, 276–280. doi: 10.1038/s41586-019-1521-8, PMID: 31485077 PMC6744281

[ref4] Belkaid Y. Hand T. W. (2014). Role of the microbiota in immunity and inflammation. Cells 157, 121–141. doi: 10.1016/j.cell.2014.03.011, PMID: 24679531 PMC4056765

[ref5] Benbara T. Lalouche S. Drider D. Bendali F. (2020). *Lactobacillus plantarum* S27 from chicken faeces as a potential probiotic to replace antibiotics: in vivo evidence. Benefic Microbes 11, 163–173. doi: 10.3920/BM2019.0116, PMID: 32131607

[ref6] Boris S. Jimenez-Diaz R. Caso J. L. Barbes C. (2001). Partial characterization of a bacteriocin produced by *Lactobacillus delbrueckii* subsp. lactis UO004, an intestinal isolate with probiotic potential. J Appl Microbiol 91, 328–333. doi: 10.1046/j.1365-2672.2001.01403.x, PMID: 11473598

[ref7] Buntyn J. O. Schmidt T. B. Nisbet D. J. Callaway T. R. (2016). The Role of Direct-Fed Microbials in Conventional Livestock Production. Annu Rev Anim Biosci 4, 335–355. doi: 10.1146/annurev-animal-022114-111123, PMID: 26667362

[ref8] Cean A. Stef L. Simiz E. Julean C. Dumitrescu G. Vasile A.. (2015). Effect of human isolated probiotic bacteria on preventing *Campylobacter jejuni* colonization of poultry. Foodborne Pathog Dis 12, 122–130. doi: 10.1089/fpd.2014.1849, PMID: 25585278

[ref9] Cerf-Bensussan N. Gaboriau-Routhiau V. (2010). The immune system and the gut microbiota: friends or foes? Nat Rev Immunol 10, 735–744. doi: 10.1038/nri2850, PMID: 20865020

[ref10] Chen X. Zhao X. Wang H. Yang Z. Li J. Suo H. (2017). Prevent effects of *Lactobacillus fermentum* HY01 on dextran sulfate sodium-induced colitis in mice. Nutrients 9:545. doi: 10.3390/nu9060545, PMID: 28587089 PMC5490524

[ref11] de Vos W. M. Tilg H. Van Hul M. Cani P. D. (2022). Gut microbiome and health: mechanistic insights. Gut 71, 1020–1032. doi: 10.1136/gutjnl-2021-326789, PMID: 35105664 PMC8995832

[ref12] Del C. A. Mayneris-Perxachs J. Fernandez-Real J. M. (2022). Bidirectional relationships between the gut microbiome and sexual traits. Am J Physiol Cell Physiol 322, C1223–C1229. doi: 10.1152/ajpcell.00116.2022, PMID: 35508190

[ref13] Diaz C. J. Casanova N. A. Fernandez M. M. (2019). Microbiota, Gut Health and Chicken Productivity: What Is the Connection? Microorganisms 7:374. doi: 10.3390/microorganisms7100374, PMID: 31547108 PMC6843312

[ref14] Hakansson A. Tormo-Badia N. Baridi A. Xu J. Molin G. Hagslatt M. L.. (2015). Immunological alteration and changes of gut microbiota after dextran sulfate sodium (DSS) administration in mice. Clin Exp Med 15, 107–120. doi: 10.1007/s10238-013-0270-5, PMID: 24414342 PMC4308640

[ref15] He Z. Ma Y. Yang S. Zhang S. Liu S. Xiao J.. (2022). Gut microbiota-derived ursodeoxycholic acid from neonatal dairy calves improves intestinal homeostasis and colitis to attenuate extended-spectrum beta-lactamase-producing enteroaggregative *Escherichia coli* infection. Microbiome 10:79. doi: 10.1186/s40168-022-01269-0, PMID: 35643532 PMC9142728

[ref16] Hu J. Chen J. Hou Q. Xu X. Ren J. Ma L.. (2023). Core-predominant gut fungus Kazachstania slooffiae promotes intestinal epithelial glycolysis via lysine desuccinylation in pigs. Microbiome 11:31. doi: 10.1186/s40168-023-01468-3, PMID: 36814349 PMC9948344

[ref17] Karami S. Roayaei M. Hamzavi H. Bahmani M. Hassanzad-Azar H. Leila M.. (2017). Isolation and identification of probiotic Lactobacillus from local dairy and evaluating their antagonistic effect on pathogens. Int J Pharm Investig 7, 137–141. doi: 10.4103/jphi.JPHI_8_17, PMID: 29184826 PMC5680649

[ref18] Khalil E. S. Abd M. M. Mustafa S. Alhelli A. M. Shokryazdan P. (2018). Probiotic Properties of Exopolysaccharide-Producing Lactobacillus Strains Isolated from Tempoyak. Molecules 23:398. doi: 10.3390/molecules23020398, PMID: 29438288 PMC6017292

[ref19] Kumar M. Kala A. Chaudhary L. C. Agarwal N. Kochewad S. A. (2022). Microencapsulated and lyophilized *Lactobacillus acidophilus* improved gut health and immune status of preruminant calves. Probiotics Antimicrob Proteins 14, 523–534. doi: 10.1007/s12602-021-09821-4, PMID: 34286420

[ref20] Li K. Luo H. Zhang H. Lan Y. Han Z. Shahzad M.. (2016). First report of Metastrongylus pudendotectus by the genetic characterization of mitochondria genome of cox1 in pigs from Tibet, China. Vet Parasitol 223, 91–95. doi: 10.1016/j.vetpar.2016.04.036, PMID: 27198783

[ref21] Li C. L. Wang J. Zhang H. J. Wu S. G. Hui Q. R. Yang C. B.. (2018). Intestinal morphologic and microbiota responses to dietary Bacillus spp. in a broiler chicken model. Front Physiol 9:1968. doi: 10.3389/fphys.2018.01968, PMID: 30705639 PMC6344408

[ref22] Linninge C. Xu J. Bahl M. I. Ahrne S. Molin G. (2019). Lactobacillus fermentum and *Lactobacillus plantarum* increased gut microbiota diversity and functionality, and mitigated Enterobacteriaceae, in a mouse model. Benefic Microbes 10, 413–424. doi: 10.3920/BM2018.0074, PMID: 30957532

[ref23] Liu S. Luo H. Wang M. Wang Q. Duan L. Han Q.. (2022). Microbiome analysis reveals the effects of black soldier fly oil on gut microbiota in pigeon. Front Microbiol 13:998524. doi: 10.3389/fmicb.2022.998524, PMID: 36160221 PMC9495606

[ref24] Liu B. Wang C. Huasai S. Han A. Zhang J. He L.. (2022). Compound probiotics improve the diarrhea rate and intestinal microbiota of newborn calves. Animals 12:322. doi: 10.3390/ani12030322, PMID: 35158646 PMC8833761

[ref25] Lutful K. S. (2009). The role of probiotics in the poultry industry. Int J Mol Sci 10, 3531–3546. doi: 10.3390/ijms10083531, PMID: 20111681 PMC2812824

[ref26] Makhloufi K. M. Carre-Mlouka A. Peduzzi J. Lombard C. van Reenen C. A. Dicks L. M.. (2013). Characterization of leucocin B-KM432Bz from *Leuconostoc pseudomesenteroides* isolated from boza, and comparison of its efficiency to pediocin PA-1. PLoS One 8:e70484. doi: 10.1371/journal.pone.0070484, PMID: 23936441 PMC3731274

[ref27] Mandal S. M. Silva O. N. Franco O. L. (2014). Recombinant probiotics with antimicrobial peptides: a dual strategy to improve immune response in immunocompromised patients. Drug Discov Today 19, 1045–1050. doi: 10.1016/j.drudis.2014.05.019, PMID: 24881782

[ref28] Nagalingam N. A. Kao J. Y. Young V. B. (2011). Microbial ecology of the murine gut associated with the development of dextran sodium sulfate-induced colitis. Inflamm Bowel Dis 17, 917–926. doi: 10.1002/ibd.21462, PMID: 21391286 PMC3058753

[ref29] Pan F. Zhang L. Li M. Hu Y. Zeng B. Yuan H.. (2018). Predominant gut *Lactobacillus murinus* strain mediates anti-inflammaging effects in calorie-restricted mice. Microbiome 6:54. doi: 10.1186/s40168-018-0440-5, PMID: 29562943 PMC5863386

[ref30] Pimentel J. D. Chan R. C. (2007). Desulfovibrio fairfieldensis bacteremia associated with choledocholithiasis and endoscopic retrograde cholangiopancreatography. J Clin Microbiol 45, 2747–2750. doi: 10.1128/JCM.00969-07, PMID: 17567792 PMC1951246

[ref31] Qin J. Li R. Raes J. Arumugam M. Burgdorf K. S. Manichanh C.. (2010). A human gut microbial gene catalogue established by metagenomic sequencing. Nature 464, 59–65. doi: 10.1038/nature08821, PMID: 20203603 PMC3779803

[ref32] Rutkowski M. Krzeminska-Fiedorowicz L. Khachatryan G. Kabacinska J. Tischner M. Suder A.. (2022). Antibacterial properties of biodegradable silver nanoparticle foils based on various strains of pathogenic bacteria isolated from the oral cavity of cats, dogs and horses. Materials 15:1269. doi: 10.3390/ma15031269, PMID: 35161213 PMC8840282

[ref33] Soumeh E. A. Cedeno A. Niknafs S. Bromfield J. Hoffman L. C. (2021). The efficiency of probiotics administrated via different routes and doses in enhancing production performance, meat quality, gut morphology, and microbial profile of broiler chickens. Animals 11:3607. doi: 10.3390/ani11123607, PMID: 34944382 PMC8697876

[ref34] Tan F. Liu G. Lau S. A. Jaafar M. H. Park Y. H. Azzam G.. (2020). Lactobacillus probiotics improved the gut microbiota profile of a *Drosophila melanogaster* Alzheimer's disease model and alleviated neurodegeneration in the eye. Benefic Microbes 11, 79–89. doi: 10.3920/BM2019.0086, PMID: 32066253

[ref35] Urata T. Kikuchi M. Hino T. Yoda Y. Tamai K. Kodaira Y.. (2008). Bacteremia caused by Desulfovibrio fairfieldensis. J Infect Chemother 14, 368–370. doi: 10.1007/s10156-008-0629-9, PMID: 18936890

[ref36] Uthaibutra V. Kaewkod T. Prapawilai P. Pandith H. Tragoolpua Y. (2023). Inhibition of skin pathogenic bacteria, antioxidant and anti-inflammatory activity of royal jelly from Northern Thailand. Molecules 28:996. doi: 10.3390/molecules28030996, PMID: 36770665 PMC9920569

[ref37] Vernocchi P. Del C. F. Putignani L. (2020). Gut microbiota metabolism and interaction with food components. Int J Mol Sci 21:3688. doi: 10.3390/ijms21103688, PMID: 32456257 PMC7279363

[ref38] Wang Y. An M. Zhang Z. Zhang W. Kulyar M. F. Iqbal M.. (2022). Effects of milk replacer-based Lactobacillus on growth and gut development of yaks' calves: a gut microbiome and metabolic study. Microbiol Spectr 10:e115522. doi: 10.1128/spectrum.01155-22, PMID: 35771011 PMC9431445

[ref39] Wang L. Zhang H. Rehman M. U. Mehmood K. Jiang X. Iqbal M.. (2018). Antibacterial activity of *Lactobacillus plantarum* isolated from Tibetan yaks. Microb Pathog 115, 293–298. doi: 10.1016/j.micpath.2017.12.077, PMID: 29305183

[ref40] Wang J. Zhu G. Sun C. Xiong K. Yao T. Su Y.. (2020). TAK-242 ameliorates DSS-induced colitis by regulating the gut microbiota and the JAK2/STAT3 signaling pathway. Microb Cell Factories 19:158. doi: 10.1186/s12934-020-01417-x, PMID: 32762699 PMC7412642

[ref41] Yu T. Guo F. Yu Y. Sun T. Ma D. Han J.. (2017). *Fusobacterium nucleatum* promotes chemoresistance to colorectal cancer by modulating autophagy. Cells 170, 548–563.e16. doi: 10.1016/j.cell.2017.07.008, PMID: 28753429 PMC5767127

[ref42] Zhang L. Chu J. Hao W. Zhang J. Li H. Yang C.. (2021). Gut Microbiota and Type 2 Diabetes Mellitus: Association, Mechanism, and Translational Applications. Mediat Inflamm 2021, 5110276–5110212. doi: 10.1155/2021/5110276, PMID: 34447287 PMC8384524

[ref43] Zhang Y. Ding Y. Mo Q. Kulyar M. F. He Y. Yao W.. (2022). Sodium butyrate ameliorates thiram-induced tibial dyschondroplasia and gut microbial dysbiosis in broiler chickens. Ecotoxicol Environ Saf 245:114134. doi: 10.1016/j.ecoenv.2022.114134, PMID: 36183428

